# Dr. F. J. Waldo on Imperial and Local Government

**Published:** 1914-07-11

**Authors:** 


### THE CORONERS' SOCIETY.

### Dr. F. J. Waldo on Imperial and Local Government.

At the annual dinner of the Coroners' Society of England and Wales, Dr. Waldo, his Majesty's Coroner for the City of London, and senior Vice-President of the Coroners' Society, in proposing the toast of "Imperial and Local Government," reviewed the part that Coroners are able to take in legislation. Speaking as a Coroner and for the Coroners' Society, they desired to associate themselves with all those who were anxious to enjoy good government, both Imperial and local, and to emphasise the fact that, though their work immediately concerned the dead, none the less they were interested in the prevention of disease, accident, and crime, which too often led to death.

In many ways members of local governing bodies, being in closer touch with the people, seemed to accomplish more solid and tangible work than Imperial legislators, and nowadays it was the fashion, very rightly, to entrust the local authorities with wider and ever-increasing delegated powers and duties. Coroners were able and willing to assist our legislators, since Coroners are brought in their Courts—not inaptly called the People's Court—into close every-day contact with the laws of the country, both Imperial and local. There was not a case coming before them which did not involve some law or other—often a good many. Coroners, too, had it in their power to effect a good deal of public good by calling attention to numerous matters, such as defects in the law of death registration, of unqualified medical practice, of Street Traffic Acts, of Children's Protection Acts, and of Building, Factory and Explosive Acts, including dangerous substances like celluloid, at present free from any legislation of a restrictive nature, and so on.

The Coroners' Society had considered at their annual meeting, and at their Council meetings during the past year or so, a Coroners Bill amending and consolidating the Law and Practice of Coroners, which had been drafted, with certain alterations and additions, on the lines of the recommendations of the Home Office Committee, as made in their Report published in 1909. Some Coroners were averse to suggesting reform, fearing, presumably, the possibility of Government giving them more than they asked for. The large majority, however, thought the present time was a fitting and proper one to make suggestions. Personally, although he was not in agreement with certain of the suggestions, still, on the whole, Dr. Waldo thought the Bill a fair and useful one. In the main, the all-important matter in the public interest was that the electing authority should continue to secure the independence of the Coroner as a public official, and that by means of liberal inducements they should attract to the service the best possible men. The Bill dealt with such important matters, among others, as the general holding of fire inquests by Coroners, the payment of fees to *all* medical witnesses—including those connected with medical institutions—the payment of all Coroners by salary in lieu of fees, and the provision for the retirement of Coroners from office with an adequate pension.

Some eight years ago Mr. Herbert Samuel, now President of the Local Government Board, in replying, when Under Secretary of State, to a similar toast, said he was strongly in favour of an extension of the powers given in the City of London Fire Inquests Act of 1888 to the whole country. He thought it was a matter to which the Coroners' Society might well give its attention, and he was sure the Home Office would be very glad indeed to have the views of the Society on the matter. Dr. Waldo expressed himself as strongly of opinion that there was no more important or useful recommendation, from a public point of view, than that urged by the Departmental Committee to the effect that inquiry should be made by *all* Coroners into the cause, circumstances, and prevention of *non-fatal*, as well as of *fatal*, fires.

